# Measuring the polymerization stress of self-adhesive resin composite cements by crack propagation

**DOI:** 10.1007/s00784-020-03391-5

**Published:** 2020-06-15

**Authors:** Felicitas Wiedenmann, Fabian Becker, Marlis Eichberger, Bogna Stawarczyk

**Affiliations:** Department of Prosthetic Dentistry, University Hospital of Munich (LMU), Goethestraße 70, 80336 Munich, Germany

**Keywords:** Crack propagation, Vickers indentation, Polymerization stress, Self-adhesive resin composite cement, Feldspar ceramic

## Abstract

**Objectives:**

To test the polymerization stress of nine self-adhesive resin composite cements (G-CEM, iCEM, Bifix SE, Maxcem Elite, PANAVIA SA, SoloCem, SmartCem 2, SpeedCEM, RelyX Unicem 2) and one glass ionomer cement (control group; Ketac Cem).

**Materials and methods:**

The crack propagation of a feldspar ceramic (*n* = 130) was determined by measuring crack lengths that originated from Vickers indentations, prior to and after the application and polymerization of the self-adhesive resin cements. Results for crack propagation were converted to polymerization stress values, and statistical analysis was performed using one-way ANOVA followed by Scheffé post hoc test.

**Results:**

SmartCem 2 presented higher stress values than iCEM, SoloCem, and Ketac Cem, while Ketac Cem showed lower values than Bifix SE, Maxcem Elite, SmartCem 2, SpeedCEM, and RelyX Unicem 2.

**Conclusions:**

Self-adhesive resin composite cements differ in their polymerization stress, which may affect the durability of the restoration. For restorations made from ceramics with lower flexural strength, such as feldspar ceramics, resin composite cement materials with less polymerization stress should be preferred.

**Clinical Relevance:**

As a high polymerization shrinkage may increase crack propagation, the determination of the polymerization stress of self-adhesive resin composite cements employed for fixing all-ceramic restorations is an important factor.

## Introduction

The fixation of all-ceramic restorations with resin composite cements allows for optimal esthetic results, while the powerful bond to the natural tooth can protect and preserve dental hard tissue, as retentive preparations become unnecessary [1]. The adhesive bond can furthermore stabilize brittle ceramic restorations, resulting in a greater resistance to external forces [2]. In addition, resin composite cements can compensate small inaccuracies of the restauration [3].

Most systems are, however, prone to error due to elaborate fastening processes, calling for a prior conditioning and pretreatment with an adhesive system. In this context, self-adhesive resin composite cements can constitute an alternative by achieving an adhesive bond between restoration and tooth without a preceding treatment of the natural tooth [4, 5]. Expect for several investigations examining different self-adhesive resin composite cements in regard to their potentially successful bonding to teeth [6–8]; few studies exist that investigate the wider implications of employing these materials.

During polymerization, the mixing of the monomeric compounds allows for the formation of a polymer by the continuous addition of active chain ends. The volume of the material subsequently decreases, as carbon double bonds are converted to carbon single bonds [9, 10]. These transitions result in polymerization stress, as polymerization shrinkage is hindered by the adjoining tooth surface and the resin itself. Polymerization stress is highly dependent on the gel point of the polymer, as within certain limits, shrinkage can be compensated due to a continued flow of the composite before reaching the gel point [11, 12]. In the post-gel phase, the polymer does, however, change its properties from a highly viscous liquid to a solid elastic phase [13], in which shrinkage cannot be compensated. Polymerization stress is thus generated within the resin composite cement, the tooth, and the adjoining surface [14, 15]. This phenomenon is affected by the composition of the material [16, 17] and the polymerization process itself [9]. In a clinical setting, polymerization stress can cause the formation of gaps and an ensuing discoloration of the restoration, the development of caries, fatigue fractures, or even a total failure of the restoration [18–21].

Over the course of time, different methods have been developed to determine the polymerization stress of resin composites, from the modified dilatometer employing a mercury column [22], and the deflecting disc technique measuring the deflection of a 0.13-mm-thin glass plate [23] to elaborate methods such as micro-computed tomography [24]. Nonetheless, each method holds different disadvantages, such as a high sensitivity to temperature variations [22] or a large variability of the results due to the fragile study setup [23]. One relatively simple yet conclusive method to measure polymerization stress is the calculation through crack propagation in brittle materials [15]. The aim of this study was to determine the polymerization stress of self-adhesive resin composite cements using Vickers indentations and crack propagation in a feldspar ceramic. The study tested the null hypothesis that self-adhesive resin composite cements do not differ in their development of polymerization stress. One glass ionomer cement was included in the study design to act as a control group.

## Materials and methods

**Fig. 1:**
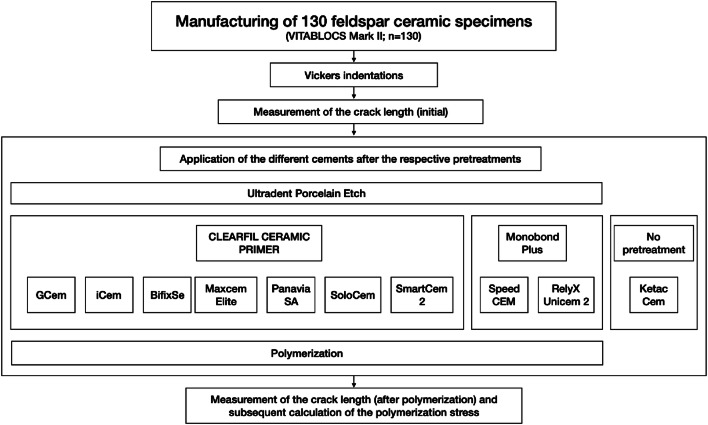
Study design

The crack propagation of a feldspar ceramic (VITABLOCS Mark II, VITA Zahnfabrik, Bad Säckingen, Germany; Table 1) was determined by measuring crack lengths that originated from Vickers indentations, prior to and after the application and subsequent polymerization of nine different self-adhesive resin composite cements and one glass ionomer cement (control group; Fig. 1, Table 2).

**Table 1:** Physical data for the feldspar ceramic VITABLOCS Mark II

Property	Values
Thermal expansion coefficient (25–500 °C)	9.4 ± 0.1 *10^-6^ • K^-1^
Density	2.44 ± 0.01 g/cm^3^
Flexural strength (Schwickerath) (ISO 6872**)**	154 ± 15 MPa
E-Modul (resonance method)	45 ± 0.5 GPa
Transformation range	780–790 °C

Source: VITA Zahnfabrik, Bad Säckingen, Germany

**Table 2:** Materials, manufacturers, compositions, and Lot No. used

Material	Manufacturer	Composition	Lot No.
VITABLOCS Mark II	VITA Zahnfabrik, Bad Säckingen, Germany	SiO_2_: 56–64, Al_2_O_3_: 20–23, Na_2_O: 6–9, K_2_O: 6–8, CaO: 0.3–0.6, TiO_2_: 0.0–0.1	7453
Ultradent Porcelain Etch	Ultradent Products, Inc., St. Louis, USA	Buffered 9% hydrofluoric acid	B863L
CLEARFIL CERAMIC PRIMER	Kuraray, Tokyo, Japan	3-Methacryloxypropyl trimethoxy silane, 10-methacryloyloxydecyl dihydrogen phosphate, ethanol	57002
Monobond Plus	Ivoclar Vivadent, Schaan, Liechtenstein	Ethanol, silane, methacrylate phosphoric ester	P20536
G-CEM	GC Europe, Leuven, Belgium	Urethane dimethacrylate (UDMA), dimethacrylate (DM), 4-MET, fluoroaluminosilicate glass, pigments, silica, initiators, stabilizers, camphorquinone, hydroperoxides	1309191
iCEM	Kulzer, Hanau, Germany	Di-, tri-, und multifunctional acrylates, initiators, stabilizers	405009
Bifix SE	VOCO, Cuxhaven, Germany	UDMA, bis-GMA, gly-DMA, phosphate-based monomers, initiators, stabilizers, glass filler, catalysts	7435
Maxcem Elite	Kerr, Orange, USA	Hydroxyethylmethacrylate (HEMA), 4-methoxyphenol (MEHQ), cumolhydroperoxid (CHPO), uncured methacrylate monomers, titanium dioxide (TiO_2_) and pigments, barium-alumina silica glass, fluoroalumina silicate glass, nano-ytterbium fluoride	4960205
PANAVIA SA	Kuraray, Tokyo, Japan	Bisphenol-A-diglycidylmethacrylate, sodium fluoride, triethyleneglycoldimethacrylate, 10-methacryloyloxydecyl-dihydrogenphosphate, hydrophobic aromatic dimethacrylate, hydrophobic aliphatic dimethacrylate, silanized barium glass filler, silanized colloidal silica, dl-camphorquinone, initiators, accelerators, catalysts	5 U0009
SoloCem	Coltene/Whaledent, Altstätten, Switzerland	UDMA, TEGDMA, 4-META, 2-hydroxyethylmethacrylate, dibenzoylperoxide, benzoylperoxide	F18973
SmartCem 2	Dentsply Sirona, Konstanz, Germany	Urethane dimethacrylate resin, ethoxylated bisphenol-A-dimethacrylate, trimethylolpropane, trimethacrylate, 2,2'-ethylendioxydiethyldimethacrylate, dimethylbenzylhydroperoxide, fluoroalumina silicate glass, catalysts, stabilizers, titanium dioxide, silicon	130430
SpeedCEM	Ivoclar Vivadent, Schaan, Liechtenstein	UDMA, triethyleneglycoldimethacrylate, polyethyleneglycoldimethacrylate, methacrylate phosphoric acid ester, dibenzoylperoxide, initiators, catalysts, barium glass, ytterbium, fluoride, silica	515015
RelyX Unicem 2	3M, Seefeld, Germany	Glass powder, mixture of mono-, di-, and tri-glycerol dimethacrylate esters of phosphoric acid, 2,2'-ethylendioxydiethyldimethacrylate, silica treated with silane, dinatriumperoxodisulfate, glass, oxides, chemicals	512279
Ketac Cem	3M, Seefeld, Germany	Silane-treated fillers, water, 2-hydroxyethylmethacrylate, silica treated with silane, 4-dimethylamino-phenethyl alcohol	537468

### Specimen preparation

**Fig. 2:**
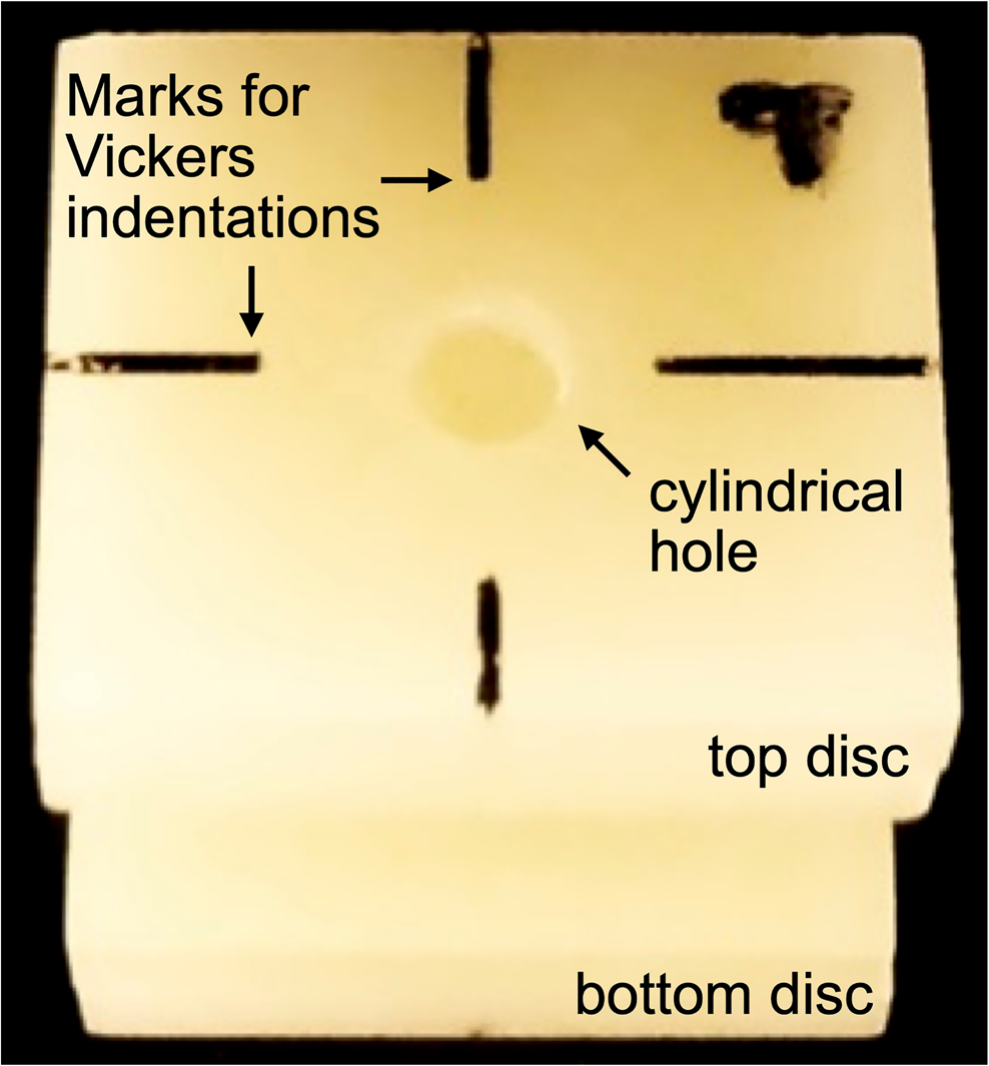
A specimen depicted with the centrally located hole of 2.5 mm and marks indicating the position of the four Vickers indentations

VITABLOCS Mark II were sliced under water cooling into 2.35-mm-thick discs using a diamond saw (Secotom 50, Struers, Ballerup, Denmark; *N* = 260). For respectively half the discs (hereafter referred to as top disc), a cylindrical hole with a diameter of 2.5 mm was manually drilled into the middle of each top disc using a turbine (K-Air Plus, KaVo, Biberbach/Riß, Germany) equipped with a diamond drill (S6856, Gebr. Brasseler, Lemgo, Germany; Fig. 2) under water cooling. The top and bottom of each disc were then polished in five steps to 4 μm under water cooling with a polishing machine (Abramin, Struers) to produce discs with a final dimension of 12 × 14 × 2 mm. Discs were annealed at 680 °C for 10 h (Arca20, Schütz Dental, Rosbach, Germany) to release possible residual stress from the processing procedure. Prior to bonding, discs were carefully cleaned with ethanol (96%, Otto Fischar). Top discs (possessing the drilled hole) and bottom discs (whole discs) were then bonded to each other employing a silicone template ensuring an exact positioning with cyanoacrylate to produce a total of 130 specimens.

### Measurement of the crack propagation

**Fig. 3:**
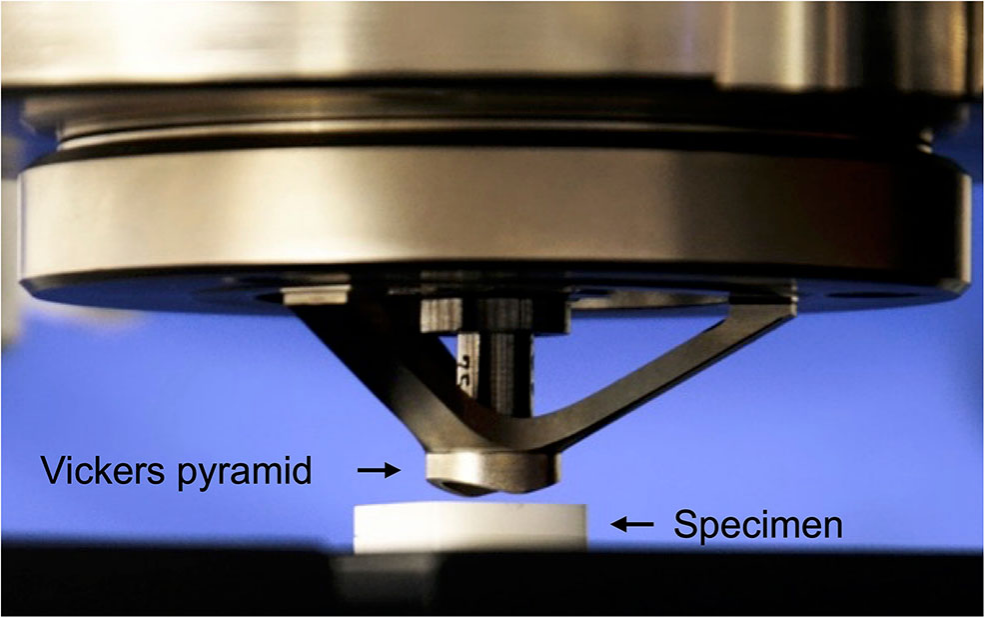
Setup for performing the Vickers indentations

**Fig. 4:**
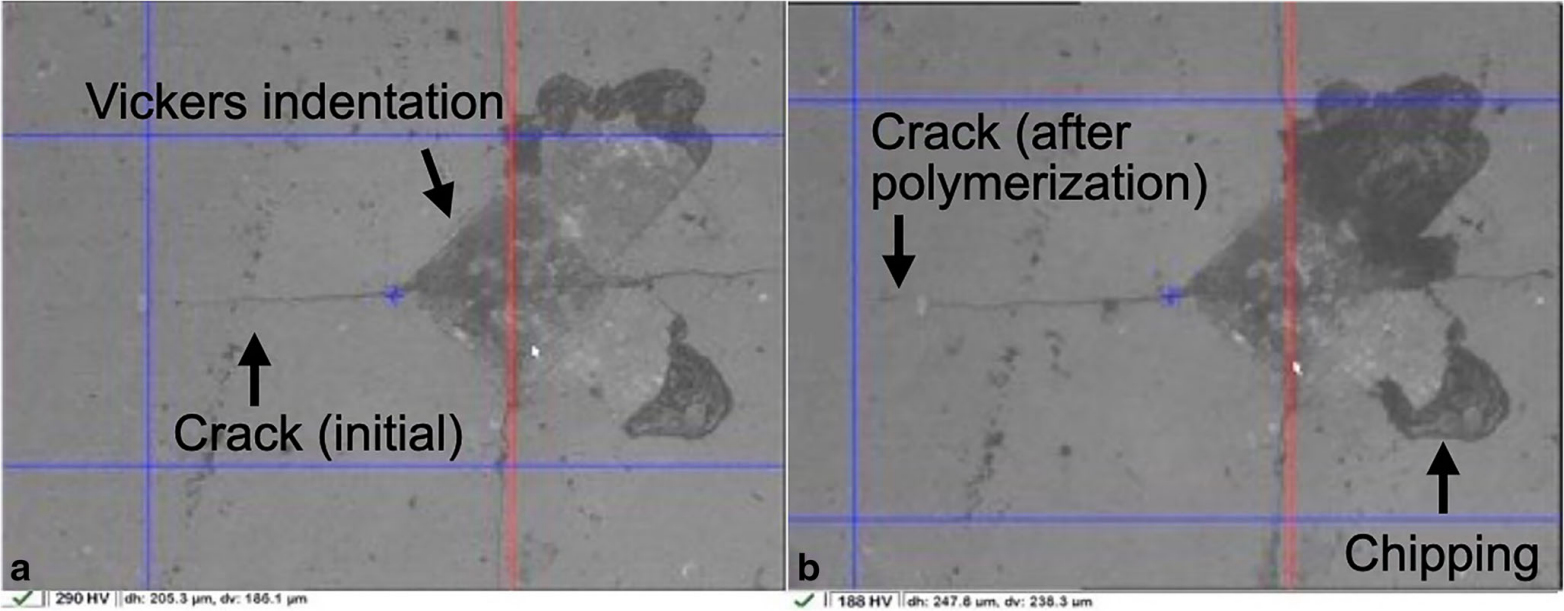
Determination of the crack propagation (initial (**a**) and after polymerization (**b**) of the self-adhesive resin composite cement RelyX Unicem 2)

To initiate crack formation in the ceramics, four Vickers indentations per specimen were performed using a load of 58.8 N for 15 s with a universal hardness testing machine (ZHU0.2/Z2.5, Zwick/Roell, Ulm, Germany; Fig. 3). The Vickers indenter was oriented to allow for a parallel alignment of the two radial cracks with the cavity edge, with each indentation centered at a distance of 570 μm from the edge of the hole (Fig. 2) [15]. The ceramic was subsequently set aside and dried in a desiccator (Duran Exsikkator, Duran Group, Mainz, Germany) at 23 °C for 24 h to allow for a slow crack growth induced by residual stress [4]. The length of the two cracks originating from each Vickers indentation was determined at a magnification of × 200 using the software of the universal hardness testing machine (TestXpert II, Version 3.2, Zwick/Roell; Fig. 4), and an average length (*c*) was calculated for each specimen.

### Application of the different cements and subsequent polymerization

To protect the Vickers indentations from contamination, they were covered with Mylar tape. The ceramic surface was pretreated with hydrofluoric acid (Ultradent Porcelain Etch, Ultradent Products, Inc., St. Louis, USA) and silane coupling agents (CLEARFIL CERAMIC PRIMER, Kuraray, Tokyo, Japan, or Monobond Plus, Ivoclar Vivadent, Schaan, Liechtenstein) strictly following the manufacturers’ recommendation (Fig. 1). The holes were then filled with increments of the nine self-adhesive resin composite cements ((1) G-CEM, GC Europe, Leuven, Belgium; (2) iCEM, Kulzer, Hanau, Germany; (3) Bifix SE, VOCO, Cuxhaven, Germany; (4) Maxcem Elite, Kerr, Orange, USA; (5) PANAVIA SA, Kuraray; (6) SoloCem, Coltene/Whaledent, Altstätten, Switzerland; (7) SmartCem 2, Dentsply Sirona, Konstanz, Germany; (8) SpeedCEM, Ivoclar Vivadent; (9) RelyX Unicem 2, 3M, Seefeld, Germany) or the glass ionomer cement (control group; Ketac Cem, 3M, Seefeld, Germany; *n* = 13 per subgroup). Specimens were covered with Mylar tape before curing of the self-adhesive resin composite cements was performed using a LED polymerization device with wavelengths between 430 and 480 nm (Elipar S10, 3M) for 60 s. The glass ionomer cement was self-cured. After polymerization, specimens were stored for 10 min before a repeated measurement of the crack length was performed.

### Determination of the polymerization stress

To calculate the polymerization stress, the equation by Yamamoto et al.:

$$ \sigma =\frac{\left[ Kc-\varPhi \kern0.75em /\kern1.5em {c}^{\prime 3/2}\ \right]}{Y{c}^{\prime 1/2}} $$

was used [15], where *σ* is the polymerization stress of the ceramic, *K*_c_ is the fracture toughness (1.37 MPa m^1/2^), *Φ* = K_c_c^3/2^, *c* is the initial crack length, *c'* is the crack length after polymerization, and *Y* is the geometrical term equal to 1.12 π^1/2^.

### Statistical analysis

Descriptive analysis followed by Kolmogorov-Smirnov and Shapiro-Wilk test for testing the violation of normal distribution was calculated. Data were analyzed using one-way ANOVA followed by Scheffé post hoc test. All *p* values below 0.05 were construed as statistically significant. Data were analyzed with SPSS version 25.0 (IBM, Armonk, NY, USA).

## Results

Initial crack lengths ranged from 209.4 ± 9.9 to 222.6 ± 11.1 μm (Table 3). After polymerization, the shortest crack length of 223.6 ± 11.5 μm was measured within the control group, while the application of RelyX Unicem 2 led to the longest crack length of 236.6 ± 9.5 μm. In line with these absolute values, the control group presented a significantly lower crack growth of only 1.0 ± 5.2 μm, while the application of RelyX Unicem 2 led to the highest crack growth (18.9 ± 7.3; *p* = 0.05). Crack propagation is displayed in Fig. 4 for one specimen initial (**a**) and after polymerization (**b**) of the self-adhesive resin composite cement RelyX Unicem 2 (× 200 magnification). The crack growth after polymerization is clearly visible on the left side of **b**; the black arrow indicates a chipping of the ceramic next to the Vickers indentation after polymerization.

**Table 3:** Descriptive statistics for the crack lengths [μm] in the ceramic

Cement	Crack length (initial)		Crack length (after polymerization)		Crack length (Δ)	
	Mean ± SD	95% CI	Mean ± SD	95% CI	Mean ± SD	95% CI
G-CEM	212 ± 13	[204; 220]	224 ± 11	[217; 230]	12.2 ± 9.1^ab^	[17.5; 6.7]
iCEM	210 ± 13	[202; 218]	220 ± 17	[210; 230]	9.9 ± 5.5^ab^	[13.1; 6.6]
Bifix SE	209 ± 10	[203; 215]	224 ± 13	[217; 232]	15.0 ± 7.2^ab^	[19.2; 10.7]
Maxcem Elite	213 ± 19	[202; 224]	228 ± 13	[220; 236]	14.7 ± 11.0^ab^	[21.2; 8.1]
PANAVIA SA	215 ± 18	[205; 226]	227 ± 14	[218; 236]	11.4 ± 7.6*^ab^	[15.9; 6.8]
SoloCem	222 ± 9	[217; 227]	230 ± 11	[223; 237]	8.0 ± 5.7^ab^	[11.3; 4.6]
SmartCem 2	216 ± 11	[209; 223]	232 ± 25	[217; 247]	15.8 ± 18.4*^ab^	[26.8; 4.8]
SpeedCEM	219 ± 16	[209; 228]	233 ± 15	[224; 242]	14.2 ± 7.7^ab^	[18.7; 9.6]
RelyX Unicem 2	218 ± 9	[212; 223]	237 ± 10	[231; 242]	18.9 ± 7.3^b^	[23.2; 14.6]
Ketac Cem (control group)	223 ± 11	[216; 229]	224 ± 12	[217; 231]	1.0 ± 5.2*^a^	[4.0; 2.2]

*Not normally distributed

^ab^Different letters present significant differences between the different cements

SmartCem 2 presented higher stress values than iCEM, SoloCem, and Ketac Cem (*p* < 0.001–0.042), while Ketac Cem showed lower values than Bifix SE, Maxcem Elite, SmartCem 2, SpeedCEM, and RelyX Unicem 2 (*p* < 0.001–0.004) (Table 4). The remaining groups were in the same value range (*p* > 0.05).

**Table 4:** Descriptive statistics for the polymerization stress [MPa] of the different cements

Cement	Polymerization stress [MPa]	
	Mean ± SD	95% CI
G-CEM	3.34 ± 2.44^abc^	[1.86; 4.82]
iCEM	2.87 ± 1.37^ab^	[2.00; 3.80]
Bifix SE	4.17 ± 1.86^bc^	[3.00; 5.30]
Maxcem Elite	4.47 ± 2.49^bc^	[2.96; 6.00]
PANAVIA SA	3.09 ± 2.05*^abc^	[1.85; 4.34]
SoloCem	2.15 ± 1.41^ab^	[1.29; 3.00]
SmartCem 2	5.90 ± 1.22^c^	[5.15; 6.64]
SpeedCEM	3.99 ± 2.12^bc^	[2.70; 5.27]
RelyX Unicem 2	4.74 ± 1.67^bc^	[3.72; 5.75]
Ketac Cem (control group)	0.39 ± 0.29*^a^	[0.22; 0.57]

*Not normally distributed

^ab^Different letters present significant differences between the different cements

## Discussion

While one previous study observed self-adhesive resin composite cements to present lower stress values than conventional resin-based cements [6], information about the development of polymerization stress in these materials is very rare. Therefore, the aim of this study was to examine the polymerization stress of nine self-adhesive resin composite cements as well as one conventional glass ionomer cement (control group) using Vickers indentations and crack propagation in a feldspar ceramic.

As the tested self-adhesive resin composite cements differed in their development of polymerization stress, the null hypothesis had to be rejected. As the development of polymerization stress depends on the filler content of the different materials [8, 16], with an inverse relationship between filler content and polymerization shrinkage existing [17], a high proportion of inorganic components should result in lower polymerization stress values. Nonetheless, an increased filler content might also adversely affect the conversion rate, as reactive groups are hindered in their free movement, while a polymerization by light activation is impaired by an increased light scattering [17]. As one previous study could demonstrate, the matrix might furthermore hold a stronger influence on stress values than the inorganic content [17]. The varying expansion behavior of the different self-adhesive resin composites could represent an additional perspective, as it might contribute to the observed findings. The initial expansion, created by an exothermic reaction and the heat generated by the curing unit, is, however, quickly compensated by the ensuing contraction early on in the curing process. A larger initial expansion in the different materials could, however, result in lower shrinkage values [8]. One further aspect is constituted by the potential later hygroscopic expansion behavior of these materials when in situ [25, 26] that can induce and reinforce crack propagation of the ceramic restoration. Self-adhesive resin cements have been observed to exhibit high values of water expansion stress [26].

In the present study, SmartCem 2 showed higher polymerization stress values than the self-adhesive resin composite cements iCEM and SoloCem. This is in line with a previous investigation reporting the highest contraction stress for SmartCem 2 in comparison with six other self-adhesive resin composite cements [26]. To draw conclusions based on this resin composite’s composition poses to be difficult, as the exact proportions of the various components are not supplied by the manufacturer. While an increase in polymerization stress can be traced back to a higher shrinkage during polymerization, it might also indicate a strong bond between ceramic and mounting material, as the crack grows until the bond dissolves [15]. Previous studies investigating the shear bond strength of SmartCem 2 observed consistently high results [27, 28]. To the authors’ best knowledge, the polymerization stress of this particular resin composite cement has not been examined beforehand. Future studies should focus on examining the two self-adhesive resin composite cements iCEM and SoloCem further, as they presented low polymerization values while allowing a self-adhesive, and thus less error prone, fixation of dental restorations. Previous studies have observed iCEM to present comparable bond strength results between ceramic and dentin after water storage as a reference etch-and-rinse resin cement [29] and to show promising results in regard to the marginal adaptation and sealing of both enamel and dentin [30]. One study investigating the hygroscopic expansion of self-adhesive resin cements after water storage did, however, report a progressive crack propagation of VITABLOCS Mark II from deep initial cracks caused during CAD/CAM processing over time [25]. SoloCem has been observed to possess a high radiopacity [31] and a high water absorbance while nonetheless yielding low levels of discoloration [32]. As it has been demonstrated that a delay of 3–5 min prior to light activation of dual-cured resin composite cements can reduce shrinkage stress and improve bond strength without significantly altering mechanical properties, this parameter should be investigated in future studies examining resin composite cements [33].

The glass ionomer cement Ketac Cem presented lower polymerization stress values than most of the tested self-adhesive resin composite cements. While this drawback has to be taken into account when choosing either a self-adhesive or a conventional cement, Ketac Cem has been shown to lead to inferior results than various self-adhesive resin composite cements in regard to long-term stability [34, 35] and bond strength [36, 37]. In the case of retentive preparations and high-strength ceramics such as monolithic zirconia, employing conventional glass ionomer cements can, however, entail many advantages [38]. As an adhesive is not required, a prolonged drainage becomes redundant. This can be especially helpful for deep posterior defects, where accessibility can be complicated. Compared with composite resins that enable the stabilization of the natural tooth structure through a strong composite-tooth bond [39], indications can, however, be limited (e.g., teeth presenting with large defects that are prone to fracture). Furthermore, glass ionomer cements are not recommended for restorative materials possessing a flexural strength lower than 350 MPa, as these restorations should be adhesively bonded to increase the overall stability and long-term outcome of the restoration [40].

As polymerization stress can lead to chipping (as depicted in Fig. 4) and the formation of marginal gaps, entailing dire consequences such as discoloration, secondary caries, fatigue fractures, and postsurgical hypersensitivity up to the total failure of a restauration [12], it represents a crucial factor that should be considered when selecting a self-adhesive resin composite cement. Although a higher polymerization stress entails many negative consequences, it might also indicate a strong bond between ceramic and mounting material, as the crack grows until the bond dissolves [15]. Further studies are warranted to investigate a possible correlation between bond strength and crack propagation. The measured stress values of the present study cannot be directly transferred to the clinical situation, as additional factors present in the oral cavity must be taken into account. An increase in ambient temperature could, for example, lead to greater shrinkage [14]. In addition, curing can differ in the clinical setting, as it depends on the different levels of transparency of a material, caused by variations in the material thickness, and the exact positioning of the polymerization device. These varying conditions can cause different zones within a resin composite cement, where the chemical and dual curing reactions take place simultaneously. While this could reduce the conversion rate, it might also reduce shrinkage [41]. As curing parameters might hold a strong influence on crack propagation, future studies should focus on investigating this aspect further by varying the duration to light exposure, light intensity, and the wavelengths of the employed polymerization devices. The determination and analyzation of the degree of conversion could in this context enhance our understanding of the observed polymerization stress results further [8]. One further limitation of the present study is constituted by the layer thickness of the fixing material, with an optimal cement gap in the clinical setting varying between 30 and 50 μm. As a reduced thickness of the fixing material should in theory reduce shrinkage, the present results need to be confirmed in future investigations expanding this novel measurement approach to imitate clinical situations as closely as possible. The configuration factor, referring to the number of bonded to un-bonded surfaces of a cavity, should also be taken into account in future study designs. While this study only examined a limited number of products, in regard to both the resin composite cements and the feldspar ceramic, future examinations should focus on testing a wider range of materials, paying special attention to their respective compositions and ensuing material properties. The choice of resin composite cement is, of course, only one factor among many that should be considered in the workflow of fixing a dental restoration, where optimal conditions allow for an enhanced treatment quality. Under laboratory conditions, crack initiation and propagation are furthermore influenced by the strength and load duration of the Vickers indentations and the distance of the indentations to the edge of the hole. Further studies are warranted to verify, whether the results obtained using this new methodology for determining the polymerization stress of self-adhesive resin composite cements can be transferred to the clinical practice.

## Conclusions

Self-adhesive resin composite cements differ in their development of polymerization stress, which may affect the durability of the restorations. For restorations made from ceramics with lower flexural strength, such as feldspar ceramics, resin composite cement materials with less polymerization stress should be preferred.

## References

[1] Edelhoff D, Ozcan M (2007). To what extent does the longevity of fixed dental prostheses depend on the function of the cement? Working group 4 materials: cementation. Clin Oral Implants Res.

[2] Abad-Coronel C, Naranjo B, Valdiviezo P (2019) Adhesive systems used in indirect restorations cementation: review of the literature. Dent J (Basel):7. 10.3390/dj7030071 10.3390/dj7030071 PMC6784471 31266163

[3] Pameijer CH (2012). A review of luting agents. Int J Dent.

[4] Ferracane JL, Stansbury JW, Burke FJ (2011). Self-adhesive resin cements - chemistry, properties and clinical considerations. J Oral Rehabil.

[5] Makkar S, Malhotra N (2013). Self-adhesive resin cements: a new perspective in luting technology. Dent Update.

[6] Frassetto A, Navarra CO, Marchesi G, Turco G, Di Lenarda R, Breschi L, Ferracane JL, Cadenaro M (2012). Kinetics of polymerization and contraction stress development in self-adhesive resin cements. Dent Mater.

[7] Kitzmuller K, Graf A, Watts D, Schedle A (2011). Setting kinetics and shrinkage of self-adhesive resin cements depend on cure-mode and temperature. Dent Mater.

[8] Spinell T, Schedle A, Watts DC (2009). Polymerization shrinkage kinetics of dimethacrylate resin-cements. Dent Mater.

[9] Ferracane JL (2005). Developing a more complete understanding of stresses produced in dental composites during polymerization. Dent Mater.

[10] Mantri SP, Mantri SS (2013). Management of shrinkage stresses in direct restorative light-cured composites: a review. J Esthet Restor Dent.

[11] Peutzfeldt A, Asmussen E (2004). Determinants of in vitro gap formation of resin composites. J Dent.

[12] Davidson CL, de Gee AJ (1984). Relaxation of polymerization contraction stresses by flow in dental composites. J Dent Res.

[13] Irie M, Tanaka J, Maruo Y, Nishigawa G (2014). Vertical and horizontal polymerization shrinkage in composite restorations. Dent Mater.

[14] Tantbirojn D, Versluis A, Pintado MR, DeLong R, Douglas WH (2004). Tooth deformation patterns in molars after composite restoration. Dent Mater.

[15] Yamamoto T, Ferracane JL, Sakaguchi RL, Swain MV (2009). Calculation of contraction stresses in dental composites by analysis of crack propagation in the matrix surrounding a cavity. Dent Mater.

[16] Baroudi K, Saleh AM, Silikas N, Watts DC (2007). Shrinkage behaviour of flowable resin-composites related to conversion and filler-fraction. J Dent.

[17] Goncalves F, Kawano Y, Braga RR (2010). Contraction stress related to composite inorganic content. Dent Mater.

[18] Kramer N, Reinelt C, Richter G, Petschelt A, Frankenberger R (2009). Nanohybrid vs. fine hybrid composite in class II cavities: clinical results and margin analysis after four years. Dent Mater.

[19] Meredith N, Setchell DJ (1997). In vitro measurement of cuspal strain and displacement in composite restored teeth. J Dent.

[20] Calheiros FC, Sadek FT, Braga RR, Cardoso PE (2004). Polymerization contraction stress of low-shrinkage composites and its correlation with microleakage in class V restorations. J Dent.

[21] Davidson CL, de Gee AJ, Feilzer A (1984). The competition between the composite-dentin bond strength and the polymerization contraction stress. J Dent Res.

[22] de Gee AJ, Davidson CL, Smith A (1981). A modified dilatometer for continuous recording of volumetric polymerization shrinkage of composite restorative materials. J Dent.

[23] Watts DC, Cash AJ (1991). Determination of polymerization shrinkage kinetics in visible-light-cured materials: methods development. Dent Mater.

[24] Sun J, Lin-Gibson S (2008). X-ray microcomputed tomography for measuring polymerization shrinkage of polymeric dental composites. Dent Mater.

[25] Kirsten M, Matta RE, Belli R, Lohbauer U, Wichmann M, Petschelt A, Zorzin J (2018). Hygroscopic expansion of self-adhesive resin cements and the integrity of all-ceramic crowns. Dent Mater.

[26] Sokolowski G, Szczesio A, Bociong K, Kaluzinska K, Lapinska B, Sokolowski J, Domarecka M, Lukomska-Szymanska M (2018) Dental resin cements-the influence of water sorption on contraction stress changes and hydroscopic expansion. Materials (Basel) 11. 10.3390/ma11060973 10.3390/ma11060973 PMC6025551 29890684

[27] Hattar S, Hatamleh M, Khraisat A, Al-Rabab'ah M (2014). Shear bond strength of self-adhesive resin cements to base metal alloy. J Prosthet Dent.

[28] Lee S-EB J-H, Choi J-W, Jeon Y-C, Jeong C-M, Yoon M-J, Huh J-B (2015). Comparative shear-bond strength of six dental self-adhesive resin cements to zirconia. Materials.

[29] Flury S, Lussi A, Peutzfeldt A, Zimmerli B (2010). Push-out bond strength of CAD/CAM-ceramic luted to dentin with self-adhesive resin cements. Dent Mater.

[30] Aschenbrenner CM, Lang R, Handel G, Behr M (2012). Analysis of marginal adaptation and sealing to enamel and dentin of four self-adhesive resin cements. Clin Oral Investig.

[31] Dukic W (2019). Radiopacity of composite luting cements using a digital technique. J Prosthodont.

[32] Liebermann A, Roos M, Stawarczyk B (2017) The effect of different storage media on color stability of self-adhesive composite resin cements for up to one year. Materials (Basel) 10. 10.3390/ma10030300 10.3390/ma10030300 PMC5503324 28772660

[33] Faria-e-Silva A, Boaro L, Braga R, Piva E, Arias V, Martins L (2011). Effect of immediate or delayed light activation on curing kinetics and shrinkage stress of dual-cure resin cements. Oper Dent.

[34] Ehlers V, Kampf G, Stender E, Willershausen B, Ernst CP (2015). Effect of thermocycling with or without 1 year of water storage on retentive strengths of luting cements for zirconia crowns. J Prosthet Dent.

[35] Luthy H, Loeffel O, Hammerle CH (2006). Effect of thermocycling on bond strength of luting cements to zirconia ceramic. Dent Mater.

[36] Abo-Hamar SE, Hiller KA, Jung H, Federlin M, Friedl KH, Schmalz G (2005). Bond strength of a new universal self-adhesive resin luting cement to dentin and enamel. Clin Oral Investig.

[37] Kim MJ, Kim YK, Kim KH, Kwon TY (2011). Shear bond strengths of various luting cements to zirconia ceramic: surface chemical aspects. J Dent.

[38] Selz CF, Strub JR, Vach K, Guess PC (2014). Long-term performance of posterior InCeram Alumina crowns cemented with different luting agents: a prospective, randomized clinical split-mouth study over 5 years. Clin Oral Investig.

[39] Luhrs AK, Guhr S, Gunay H, Geurtsen W (2010). Shear bond strength of self-adhesive resins compared to resin cements with etch and rinse adhesives to enamel and dentin in vitro. Clin Oral Investig.

[40] Stawarczyk B, Beuer F, Ender A, Roos M, Edelhoff D, Wimmer T (2013). Influence of cementation and cement type on the fracture load testing methodology of anterior crowns made of different materials. Dent Mater J.

[41] Arrais CAG, Giannini M, Rueggeberg FA, Pashley DH (2007). Effect of curing mode on microtensile bond strength to dentin of two dual-cured adhesive systems in combination with resin luting cements for indirect restorations. Oper Dent.

